# Serum metabolomic characterization of PLA2G6-associated dystonia–parkinsonism: A case-control biomarker study

**DOI:** 10.3389/fnins.2022.879548

**Published:** 2022-08-11

**Authors:** Chen Chen, Min-Min Lou, Yi-Min Sun, Fang Luo, Feng-Tao Liu, Su-Shan Luo, Wen-Yuan Wang, Jian Wang

**Affiliations:** ^1^State Key Laboratory of Medical Neurobiology, Department of Neurology and National Research Center for Aging and Medicine, Huashan Hospital, Fudan University, Shanghai, China; ^2^State Key Laboratory of Medical Neurobiology, National Center for Neurological Disorders, Huashan Hospital, Fudan University, Shanghai, China; ^3^Interdisciplinary Research Center on Biology and Chemistry, Shanghai Institute of Organic Chemistry, Chinese Academy of Sciences – University of Chinese Academy of Sciences, Shanghai, China

**Keywords:** PLA2G6, metabolomics, early-onset parkinsonism, long chain fatty acids, serum biomarkers

## Abstract

**Introduction:**

Phospholipase A2 Group VI (PLA2G6), encoding calcium-independent phospholipase A_2_, has been isolated as the gene responsible for an autosomal recessive form of early-onset Parkinson’s disease (namely, PARK14). Compared to idiopathic Parkinson’s disease (iPD), PARK14 has several atypical clinical features. PARK14 has an earlier age at onset and is more likely to develop levodopa-induced dyskinesia. In iPD, serum metabolomics has observed alterations in several metabolic pathways that are related to disease status and clinical manifestations. This study aims to describe the serum metabolomics features of patients with PARK14.

**Design:**

This case-control biomarker study tested nine patients diagnosed with PARK14. Eight age and sex-matched healthy subjects were recruited as controls. To evaluate the influence of single heterozygous mutation, we enrolled eight healthy one-degree family members of patients with PARK14, two patients diagnosed with early-onset Parkinson’s disease (EOPD) who had only a single heterozygous PLA2G6 mutation, and one patient with EOPD without any known pathogenic mutation.

**Methods:**

The diagnosis of PARK14 was made according to the diagnostic criteria for Parkinson’s disease (PD) and confirmed by genetic testing. To study the serum metabolic features, we analyzed participants’ serum using UHPLC-QTOF/MS analysis, a well-established technology.

**Results:**

We quantified 50 compounds of metabolites from the serum of all the study subjects. Metabolites alterations in serum had good predictive accuracy for PARK14 diagnosis (AUC 0.903) and advanced stage in PARK14 (AUC 0.944). Of the 24 metabolites that changed significantly in patients’ serum, eight related to lipid metabolism. Oleic acid and xanthine were associated with MMSE scores. Xanthine, L-histidine, and phenol correlated with UPDRS-III scores. Oleic acid and 1-oleoyl-L-alpha-lysophosphatidic acid could also predict the subclass of the more advanced stage in the PLA2G6 Group in ROC models.

**Conclusion:**

The significantly altered metabolites can be used to differentiate PLA2G6 pathogenic mutations and predict disease severity. Patients with PLA2G6 mutations had elevated lipid compounds in C18:1 and C16:0 groups. The alteration of lipid metabolism might be the key intermediate process in PLA2G6-related disease that needs further investigation.

## Introduction

Phospholipase A2 Group VI (PLA2G6), encoding calcium-independent phospholipase A2, has been isolated as the gene responsible for an autosomal recessive form of early-onset Parkinson’s disease (PARK14; Clarimon and Kulisevsky, 2013). PLA2G6 is also the causative gene of infantile neuroaxonal dystrophy (INAD; Morgan et al., 2006) and neurodegeneration with brain iron accumulation (NBIA-2B; Gregory et al., 2008). Thus, these three different neurodegenerative disorders caused by PLA2G6 mutations are collectively called PLA2G6-associated neurodegeneration (PLAN; Magrinelli et al., 2021).

Genetic studies of Parkinson’s disease (PD) have revealed a number of pathogenic genes. PLA2G6-related young-onset dystonia–parkinsonism is listed as PARK14 (Clarimon and Kulisevsky, 2013). PARK14 shares some common clinical features with iPD, for instance, parkinsonism that dramatically responds to levodopa. Nevertheless, there are more atypical manifestations in patients with PARK14 (Magrinelli et al., 2021). Dystonia and levodopa-induced dyskinesia appear severer and earlier in PARK14. Cerebellar signs and pyramidal features might also point to PARK14 (Magrinelli et al., 2021).

There is growing evidence that suggests PLAN is a continuous spectrum of disease (Erro et al., 2017). INAD has an age at onset of 6 to 36 months and is characterized by psychomotor regression, intellectual disability, axonal neuropathy, spastic tetraparesis, and optic atrophy. Due to its rapid progression, patients with INAD mostly die before the first decade of life. NBIA-2B usually manifests at preschool age with cerebellar, pyramidal, or dystonic movement disorders and mental retardation. Hyperintensity of bilateral basal ganglia in magnetic resonance imaging (MRI)-T2 scan is the representative neuroradiological finding in NBIA-2B, which indicates iron deposit. The kernel manifestation of PARK14 is a dystonia–parkinsonism syndrome with or without concomitant ataxia, spastic paraplegia, and cognitive impairment. The age at onset of PARK14 is often in the second-to-third decades of life. Both child-onset PLAN and PARK14 showed the pathology of α-synuclein and tau accumulation in the neuron in several former studies (Paisan-Ruiz et al., 2012), which is also the most widely recognized pathological change in idiopathic Parkinson’s disease (iPD).

The nervous system is enriched in lipids and contains a more diverse lipid composition than other tissues to maintain neuronal functions. As a member of the phospholipase A2 (PLA2) family, PLA2G6 hydrolyzes the sn-2 ester bonds of phospholipids, generating free fatty acids (FFAs) and lysophospholipids (Burke and Dennis, 2009). A study by Mori et al. (2019) revealed that PLA2G6 loss results in acyl-chain shortening in phospholipids, which affects ER homeostasis and neurotransmission and promotes α-synuclein aggregation. Another study by Sun et al. (2021) discovered that PLA2G6 can preferentially hydrolyze peroxidized phospholipids and eliminate the ferroptotic 15-hydroperoxy (Hp)-arachidonoyl-phosphatidylethanolamine (15-HpETE-PE) death signal. Thus, PLA2G6 dysfunction can cause cell death and α-synuclein aggregation via abnormal metabolism of phospholipids.

Currently, efficient disease-modifying therapy remains a challenge in iPD treatment. But for INAD and NBIA-2B, there are small molecule therapies and gene therapies under investigation. Recognizing PLA2G6-related disease may, therefore, bring therapeutic options to patients. PLA2G6 is an intracellular phospholipase. The enzyme activity of PLA2G6 cannot be measured in serum, making the evaluation of PLA2G6 enzyme function more difficult in clinical practice. Also, it remains controversial whether PARK14-associated PLA2G6 mutations reduced phospholipase activity (Engel et al., 2010; Chiu et al., 2017). Measuring enzyme activity might not be enough to figure out PARK14-associated PLA2G6 mutations. Therefore, potential and novel biomarkers are required for PLA2G6-mediated parkinsonism.

Here we report a comprehensive serological metabolomic profiling using a well-characterized cohort of PLA2G6-associated young-onset dystonia–parkinsonism patients and matched controls with the aim to describe metabolic features and identify potential biomarkers for the PLA2G6 mutations and severity of clinical symptoms.

## Materials and methods

### Patient characterization

Two hundred eighty-two patients with a diagnosis of PD with an age of onset <50 years or with positive family history were recruited from the movement disorders outpatient clinic of the Fudan University Affiliated Huashan Hospital, Shanghai, China. The diagnostic criteria used for PD were the UK PD Society Brain Bank Clinical Diagnostic Criteria (Hughes et al., 1992) (for patients recruited before 2016) and the 2015 Movement Disorder Society (MDS) Clinical Diagnostic Criteria for PD (Postuma et al., 2015) (for patients recruited from 2016 on). All diagnoses were made by three senior movement disorder specialists. Family pedigrees were drawn for all patients. All participants provided written informed consent prior to participating in this study. After obtaining signed informed consent from all participants, venous blood samples for biochemical and molecular genetic studies were collected.

The age of onset of PD was considered the age at which the first motor symptoms manifested. Each patient was carefully assessed for motor symptoms, non-motor features, and cognitive status through a face-to-face interview. The severity of motor phenotype was investigated with the modified Hoehn and Yahr stage (H&Y) and Unified Parkinson’s Disease Rating Scale (UPDRS)-III scale (MDS-UPDRS-III for visits after 2019). We used conversion formulas to convert all the MDS-UPDRS-III scores to UPDRS-III scores in the analysis (Goetz et al., 2012). A rough description of disease progression was calculated as UPDRS-III scores divided by disease duration. Information on dopaminergic medications was retrieved, and the levodopa equivalent doses (LEDDs) were calculated (Tomlinson et al., 2010).

The patients underwent neuropsychological examination while on their routine medications. None of the patients were treated with benzodiazepines, neuroleptics, or antidepressants. Global cognition was evaluated using the Mini-Mental State Examination (MMSE) (Katzman et al., 1988).

### Molecular genetic analysis and classification of patient groups

Deoxyribonucleic acid (DNA) from the peripheral blood leucocytes of the 282 patients with PD was enriched for target regions by performing solution-based hybridization capture. High-throughput sequencing was carried out using an Illumina HiSeq 2000 Sequencer (Illumina, San Diego, CA, United States). A panel of 254 PD-related genes including PLA2G6 was designed (Supplementary Table 1). The genetic analysis was carried out by target sequencing and multiple ligation-dependent probe amplification (MLPA) as previously reported (Wang et al., 2017). The variants on all suspected variants were further confirmed by Sanger sequencing using the standardized procedure.

The variants on the exotic and splicing sites (within 100 bp of a splice junction) were analyzed. Mutation reads of <5 and mutation frequency of <30% were filtered out. Synonymous variants and the variants with an allelic frequency higher than 1% in 1000Genome, ESP6500, and in-house databases were excluded. HGMD professional (RRID:SCR_001888) and ClinVar^1^ database were used to detect the pathogenic level of the variants. The variants not included in the database were further classified by the American College of Medical Genetics and Genomics and the Association for Molecular Pathology (ACMG) Standards using the terms “pathogenic,” “likely pathogenic,” “uncertain significance (VUS),” “likely benign,” and “benign” (Richards et al., 2015).

For the metabolomics assay, we selected all patients (*n* = 9), who carried homozygous or compound heterozygous of the PLA2G6 mutations with ACMG classification as “pathogenic” or “likely pathogenic” as the *PLA2G6 Group.* We also recruited eight age and gender-matched healthy subjects as the *Healthy Control Group* in the assay. To observe the metabolic features in healthy subjects with single heterozygous PLA2G6 mutation, we collected serum from eight healthy one-degree family members of the patients with PLA2G6 mutations (*Single Heterozygous Control Group*). Also, we harvested serum from two patients diagnosed with early-onset Parkinson’s disease (EOPD) who had only a single heterozygous PLA2G6 mutation and one patient with EOPD without any known pathogenic mutation in disease-causing genes including PLA2G6 as the *EOPD Group*. Demographic and clinical data in the four groups were compared using a *t*-test, Mann–Whitney *U* test, one-way ANOVA, or chi-square test as appropriate and according to data distribution.

### Human primary skin fibroblasts culturing

PLA2G6 is an intracellular phospholipase. For enzyme activity evaluation, we use cell lysates derived from primary cultured human dermal fibroblasts. Fibroblasts were generated under the supervision of the ethics committee of Huashan Hospital of Fudan University. Briefly, human skin biopsies were conducted with a-2 mm skin punch from the ankle of healthy donors and patients with PD. The biopsies were conducted by certificated physicians. The skin samples were then sterilized, trypsinized, and minced to <0.5 mm size pieces. Those skin pieces were plated into a 24-well plate underneath a piece of the coverslip and fed with human fibroblast medium (15% FBS, 1% GlutaMAX, 1% non-essential amino acids, 55 mM β-mercaptoethanol, and 1% anti-anti in α-MEM basal medium) supplemented with 10 ng/ml of bFGF (PeproTech, 100-18B) for up to 1 month. Fibroblasts were harvested when the outgrowth fibroblasts covered the whole well bottom. Fibroblasts were passed one or two times before enzyme activity analysis.

### Measurement of PLA2G6 enzyme activity

A modified PLA2 assay kit (Cayman and Abcam) was used as previously described (Zhou et al., 2016a). Briefly, each sample of live human primary skin fibroblasts was homogenized using a cold lysis buffer (10 mM Tris–HCl, pH 7.0, 300 mM sucrose, 0.5% Triton X-100). To identify the specific activity of Ca^2+^-independent PLA2G6, the assay buffers were modified to contain no Ca^2+^. To assess the catalytic activity of PLA2G6, the cells were homogenized and treated with 10 mM EGTA for 10 min, which is known to directly displace inhibitory calmodulin and fully activate PLA2G6. For the analysis of PLA2G6 activation by store depletion, live cells were pretreated with thapsigargin (TG) (5 μM for 10 min) before homogenization, and homogenates were not treated with 10 mM EGTA. The specificity of PLA2G6 activity in both cases was confirmed by its inhibition with S-BEL (25 μM for 10 min), a chiral-specific suicidal substrate (Jenkins et al., 2002) that discriminates PLA2G6 from all other phospholipases. PLA2G6 activity in each sample was assayed (in triplicates) by incubating the samples with the substrate, 1-hexadecyl-2-arachidonoylthio-2-deoxy-sn-glycero-3-phosphorylcholine for 1 h at room temperature in a modified Ca^2+^-free assay buffer (10 mM HEPES, pH 7.4, 300 mM NaCl, 60% glycerol, 8 mM Triton X-100, 4 mM EGTA, and 2 mg/ml of bovine serum albumin). The reaction was stopped and the generation of free thiols was visualized by the addition of DTNB [5,5′-dithiobis-(2-nitrobenzoic acid)] for 5 min: the absorbance was determined at 405 nm using a standard microplate reader. In calculations of specific PLA2G6 activity, a value of 10 mM^–1^ was used as an extinction coefficient for DTNB at 405 nm. The activity of PLA2G6 was expressed in nM min^–1^ per mg of protein. Statistical evaluation of the enzyme activity results was carried out using a *t*-test.

### Sample preparations and metabolomics analysis

#### Reagents and materials

LC-MS grade water (H_2_O), acetonitrile (ACN), methanol (MeOH), 0.1% formic acid (FA) in water, and 0.1% FA in ACN were purchased from Honeywell (Muskegon, MI, United States). Ammonium fluoride (NH4F) was purchased from Sigma–Aldrich (St. Louis, United States) and dissolved in the LC-MS grade water prior to use.

#### Serum sample collection

The serum samples from all participants were collected. All of the participants were in an overnight fasting state and 5 ml of peripheral venous blood was taken in the morning. The blood was then allowed to clot for 30 min at 37°C water batch and followed by centrifugation at 3,000 rpm for 15 min. Then, the serum supernatant was taken, immediately frozen in liquid nitrogen, and stored at –80°C until further analyses.

#### Sample preparation

The serum samples were thawed on ice. Then, 50 μL of serum sample was placed in a 96-well plate and mixed with 150 μL of cold MeOH using Bravo liquid handling system (Agilent Technologies, United States), followed by vortexing for 30 s and incubation for 2 h at –20°C to precipitate proteins. The 96-well plate was then centrifuged at 4,000 rpm for 20 min at 4°C. The resulting supernatants were transferred to LC-MS vials and stored at –80°C until the UHPLC-QTOF/MS analysis. The quality control samples (QC) were prepared by pooling aliquots (10 μL) of each sample.

#### UHPLC-QTOF/MS analysis

The MS data acquisition is according to the previous protocol (Zhou et al., 2016b). The serum samples were randomly injected for the UHPLC-QTOF/MS analysis. Blank samples (75% ACN in water) and QC samples were injected every eight samples during acquisition.

The LC-MS analysis was performed utilizing a UHPLC system (Nexera UHPLC LC-30A, SHIMADZU Technologies, Japan) coupled with a quadrupole time-of-flight (QTOF) mass spectrometer (AB 6600 TripleTOF, SCIEX, Canada) in positive (+ ESI) and negative modes (–ESI), respectively. Samples (2 μL) were eluted on Waters ACQUITY UPLC BEH Amide column (100 mm × 2.1 mm, 1.7 μm particle size) at 25°C with a flow rate of 0.5 mL/min. Mobile phase A was ultrapure water containing 25 mM ammonium hydroxide (NH4OH) and 25 mM ammonium acetate (NH4OAc) and mobile phase B was pure ACN. Samples were eluted according to the following gradient: 0–0.5 min, 95% B; 0.5–7 min, 95% B to 65% B; 7–8 min, 65% B to 40% B; 8–9 min, 40% B; 9–9.1 min, 40% B to 95% B; 9.1–12 min, 95% B. The MS was operated in the information-dependent acquisition (IDA) mode with an acquisition rate of 4 spectra/s, and the TOF mass range was set as m/z 60–1200 Da. The source parameters were set as follows: GAS1, 60; GAS2, 60; CUR, 30; TEM, 650°C; and ISVF, 5000 V (positive) and –4000 V (negative). The product ion scan parameters were set as m/z 25–1200, collision energy: 30 eV, and accumulation time: 30 ms.

#### Data preprocessing and annotation

MS raw data (.wiff) were converted to the mzXML format using ProteoWizard software^2^. R package XCMS was used to extract peaks (Smith et al., 2006). The main parameters of XCMS processing were set as follows: mass accuracy in peak detection = 25 ppm; peak width = (5, 30); snthresh = 3; bw = 5; and minfrac = 0.5. A data matrix consisting of retention time, mass-to-charge ratio, and peak intensity was generated by XCMS. All metabolic peaks with a value of <80% in all quality control (QC) samples were excluded. The support vector regression normalization method based on QC samples (Zhou et al., 2016b) was used to normalize the original data. Peaks with a relative standard deviation of >30% in QC samples were removed from the peak table. The peak table and mgf format MS2 files (converted by ProteoWizard) were uploaded to the MetDNA websever (Shen et al., 2019) for metabolite identification.

### Statistical analysis

**FIGURE 1:**
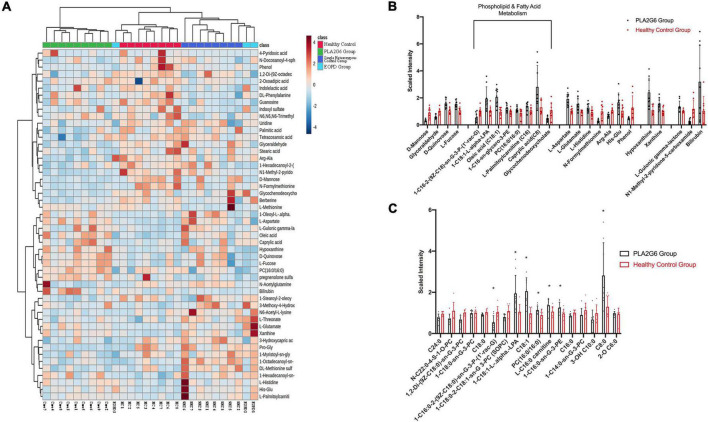
Serum metabolites alterations in PLA2G6 mutation carriers. **(A)** The heat map of clustering analysis in PLA2G6 Group (green), Healthy Control Group (red), Single Heterozygous Control Group (dark blue), and early-onset Parkinson’s disease (PD) Group (light blue); **(B)** Comparative mean scaled intensities for the 24 significantly different metabolites between early-stage PD (shaded) and age-matched controls (unshaded), showing standard error of the mean. Highlighted are the eight metabolites associated with phospholipid and fatty acid metabolism; **(C)** Comparative mean scaled intensities for all the fatty acid metabolites between early-stage PD (shaded) and age-matched controls (unshaded), sorted by the length of the carbon chain. Scaled intensity: peak intensity of each compound is normalized by median. *With statistical significance (*p* < 0.05).

Principal component analysis (PCA) and orthogonal projections to latent structures discriminant analysis (OPLS-DA) plots were generated using MetaboAnalyst 5.0. For data visualization in Figures 1B,C, the peak intensity of each compound was normalized by a median. For other data analyses, concentration data were mean-centered and divided by the range of each variable (data distribution before and after normalization and range scaling is shown in Supplementary Figure 3). Normalized data were analyzed using SPSS (Version 22.0. Armonk, NY, United States: IBM Corp.) and R version 3.2.1 with data appropriate tests. Group differences in categorical variables (e.g., gender) were assessed with the chi-square tests. Group comparisons of all other clinical characteristics (e.g., age, UPDRS-III score, and LEDD) and relative levels of different indexes were performed using Student’s *t*-tests if continuous variables follow a normal distribution, otherwise, the Mann–Whitney U test was used. The issue of multiple comparisons is handled using a common false discovery rate (FDR) approach. Relationships between metabolites and clinical measurements were explored using Spearman’s correlation coefficient. The correlation results are shown in the form of a matrix. Receiver operating characteristic (ROC) curves were constructed and the areas under the ROC curves (AUCs) were calculated to evaluate the characteristics of metabolites that could be sensitive biomarkers to identify the presence or absence of PLA2G6 mutations as well as the severity of the disease. Statistical significance was set at *p* < 0.05. For multiple testing, statistical significance was set at *p* < 0.05 after FDR correction.

## Results

### Mutations in the PLA2G6 gene

Nine out of 282 patients (3.19%) carried homozygous or compound heterozygous PLA2G6 mutations. Eight patients were compounds heterozygous and one patient was homozygous (c.991G > T). In the PLA2G6 carriers’ group, 12 mutations were found. Among them, three different PLA2G6 mutations were already reported to be associated with EOPD (c.1427 + 1G > A, c.1495G > A, c.991G > T). Nine novel PLA2G6 mutations were not previously reported either in EOPD or other neurodegeneration diseases. The three of them were novel missense changes at amino acid residues where different missense changes have been reported in INAD cases or NBIA cases before (c.1945C > T, c.1511C > T, c.668C > T). The PLA2G6 variants evaluated with ACMG guidelines are outlined in Table 1. All of the 12 mutations were evaluated as likely pathogenic or pathogenic. The two patients in the EOPD Group carried c.991G > T heterozygously.

**TABLE 1:** Evaluation of mutations discovered in the PLA2G6 Group.

Mutation (NM_003560)	Patient	Cosegregation phase	Frequency gnome AD	Prediction					Reference			ACMG interpretation
	
				SIFT	Polyphen2	Mutation taster	Conservation	Human splicing finder	Clinvar	HGMD	Publication	
c.1495G > A (p.Ala499Thr)	Case1	In *trans* with p. Thr10fs	6.46E-5	Damaging	Probably damaging	Disease causing	Conserved	–	Likely pathogenic	EOPD	EOPD, INAD	Likely pathogenic
c.28dupA (p. Thr10fs)	Case1	In *trans* with p.Ala499Thr	No result					–	No result	No result	No result	Pathogenic
c.1945C > T (p.Arg649Cys)	Case2	In *trans* with p.Gln173Ter	3.233E-5	Damaging	Probably damaging	Disease causing	Conserved	–	VUS	p.Arg649His in atypical NAD	No result	Likely pathogenic
c.517C > T (p.Gln173Ter)	Case2	In *trans* with p.Arg649Cys	No result			Disease causing	Conserved	–	Pathogenic	No result	No result	Pathogenic
c.1665C > G (p.Tyr555Ter)	Case3	In *trans* with p.Asp331Tyr	No result	Tolerated	Possibly damaging	Disease causing	substitutions	–	No result	No result	No result	Pathogenic
c.991G > T (p.Asp331Tyr)	Case3,5,6,7,8, two single heterozygous EOPD patients	Homozygous or in *trans* with p.Tyr555Ter, c.1427 + 1G > A, p.Tyr254Ter	9.69E-5	Damaging	Probably damaging	Disease causing	Conserved	–	Pathogenic	EOPD	EOPD	Likely pathogenic
c.1645T > A (p.Phe549Ile)	Case4	In *trans* with p.Ser504Leu	No result	Deleterious	Probably damaging	Disease causing	Conserved	–	No result	No result	No result	Likely pathogenic
c.1511C > T (p.Ser504Leu)	Case4	In *trans* with p.Phe549Ile	No result	Deleterious	Possibly damaging	Disease causing	Conserved	–	No result	No result	p.Ser504Arg in NBIA	Likely pathogenic
c.1427 + 1G > A	Case5	In *trans* with p.Asp331Tyr	No result		–	Disease causing	Conserved	Acceptor affected (ΔCV – 31.56%)	Pathogenic	INAD	EOPD, NBIA, INAD	Pathogenic
c.762C > A (p.Tyr254Ter)	Case6,7	In *trans* with p.Asp331Tyr	No result	–	–	Disease causing	Conserved	–	No result	No result	No result	Pathogenic
c.1915G > A (p.Ala639Thr)	Case9	In *trans* with p.Pro223Leu	No result	Damaging	Probably damaging	Disease causing	Conserved		No result	No result	No result	Likely pathogenic
c.668C > T (p.Pro223Leu)	Case9	In *trans* with p.Ala639Thr	No result	Damaging	Probably damaging	Disease causing	Conserved		No result	p.Pro223Gln in INAD	No result	Likely pathogenic

EOPD, early-onset Parkinson’s disease; INAD, infantile neuroaxonal dystrophy; NBIA, neurodegeneration with brain iron accumulation; ACMG, 2015 guidelines provided by the American College of Medical Genetics and Genomics and Allocation; HGMD, The human gene mutation database, http://www.hgmd.cf.ac.uk/ac/index.php; gnomAD, Genome Aggregation Database, https://gnomad.broadinstitute.org/; ClinVar, https://www.ncbi.nlm.nih.gov/clinvar/. ClinVar aggregates information about genomic variation and its relationship to human health.

### Demographic and clinical characterization of study subjects

Nine patients with PLA2G6 mutations and eight healthy controls were enrolled. Demographic data and clinical data are shown in Table 2. The ratio of gender and age at the time of blood collection were similar in the two study groups, while the education years of the PLA2G6 Group were significantly shorter than healthy controls (*p* = 0.003). The average PD disease duration was 31.44 months for the PLA2G6 mutation carriers. The mean UPDRS-III score was 42.44, and the average H&Y stage was 3.22. The mean progression of patients with PLA2G6 mutation was 1.72 scores per month. The average MMSE score for the PLA2G6 mutation carriers was 25, while for healthy controls was 28.8 (*p* = 0.004). There was no statistical difference in clinical features between the *PLA2G6 Group* and the *EOPD Group*.

**TABLE 2:** Demographic and clinical characterization of study subjects.

	PLA2G6 group	EOPD group	Single heterozygous mutation healthy group	Healthy control group	*P*-value
No. of subjects	9	3	8	8	–
Age at exam, y	28.89 ± 4.08	31.67 ± 1.53	54.0 ± 4.93	30.38 ± 4.40	0.543^a^
Gender (M/F)	7/2	2/1	2/6	7/1	1.000^a^
Age at onset, y	25.89 ± 3.95	24.0 ± 9.54	–	–	0.620^b^
Education, y	11.67 ± 3.57	10.0 ± 1.73	–	15.21 ± 1.90	0.012^a^
Disease duration, month	31.44 ± 14.22	74.67 ± 74.77	–	–	0.482^c^
H&Y stage	3.22 ± 0.97	2.67 ± 0.58	–	–	0.482^c^
UPDRS-III score (off)	42.44 ± 12.56	41.0 ± 15.62	–	–	0.873^b^
Disease progression, points per month	1.72 ± 1.09	0.80 ± 0.38	–	–	0.197^b^
MMSE	25.00 ± 6.07	27.33 ± 3.78	–	28.80 ± 1.23	0.004^a^*
Levo-Dopamine (L-Dopa) equivalent dose, mg	676.87 ± 290.99	425.40 ± 176.21	–	–	0.281^b^
TG activated iPLA enzyme activity, nM/min/mg	2.74 ± 0.92	–	–	8.27 ± 2.36	0.003^d^*

EOPD early-onset Parkinson’s Disease.

^a^Compared with one-way ANOVA or Chi-square test in all the groups except the Single heterozygous mutation Healthy Control Group.

^b^Compared with *t*-test between PLA2G6 Group and EOPD Group.

^c^Compared with U-test between PLA2G6 Group and EOPD Group.

^d^Compared with U-test between PLA2G6 Group and Healthy Control Group. *p < 0.05.

### Ca^2+^ -independent PLA2G6 enzyme activity changes

Five of the patients in the PLA2G6 Group (Case1,2,3,6,8) and three of the healthy controls donated their skin fibroblasts. Summary data of PLA2G6 enzyme activity in skin samples were shown in Supplementary Figure 1A. Mean values of the TG-induced Ca^2+^ storage-dependent activation of PLA2G6 in the five patients and three controls are shown in Table 2. We found that the activation of PLA2G6 by TG-induced Ca^2+^ store depletion seen in control donors (Csutora et al., 2006) was significantly impaired in the cells from PLA2G6 carriers (Supplementary Figure 1C), while the baseline catalytic activity of PLA2G6 was similar (Supplementary Figure 1B). The specificity of PLA2G6 activity in both cases was confirmed by its inhibition with S-BEL (Jenkins et al., 2002), a chiral-specific suicidal substrate that discriminates PLA2G6 from all other phospholipases.

### Serum metabolites alterations in PLA2G6 mutation carriers

To select the metabolites species that were more reflective of PLA2G6 mutation carriers, we compared metabolomic profiles in the patients (*n* = 9), patients with EOPD with only a single heterozygous mutation and no disease-causing mutation (altogether *n* = 3), healthy subjects with a single heterozygous mutation (*n* = 8) and healthy controls (*n* = 8). PCA of post-QC scaled metabolite data indicates that the PLA2G6 Group and the Healthy Control Group had distinct metabolic profiles (Supplementary Figure 2A). In cross-validated orthogonal partial least squares discriminant analysis (OPLS-DA, Supplementary Figure 2B), all of the four groups showed a significant difference in the distribution of metabolites. The three patients in the EOPD Group are located exactly between the PLA2G6 Group and the Healthy Control Group. Healthy subjects with a single heterozygous mutation were found on the same side as the Healthy Control Group.

Comparative results (normalized by range scaling) are presented in the heat map (Figure 1A). The two patients with EOPD with only a single heterozygous mutation were located beside the healthy subjects with a single heterozygous mutation (*Single Heterozygous Control Group*), and one patient with EOPD with no disease-causing gene mutation was located alongside the *Healthy Control Group.* Cluster analysis confirmed that compared with the PLA2G6 Group, subjects with only a single heterozygous mutation had more similar metabolic patterns to subjects with no PLA2G6 mutation, regardless of the diagnosis.

To investigate the impact of PLA2G6 mutation in patients, we calculated the difference between the PLA2G6 Group and Healthy Control Group thereinafter. We detected 50 known compounds in metabolites profiling, in which 24 metabolites changed significantly in patients’ serum after FDR correction (Table 3); 10 changed more than 2-folds (see volcano plot in Supplementary Figure 2C). To visualize the results, concentration data shown in Figures 1B,C were normalized by median normalization. In the negative mode, 1-hexadecanoyl-sn-glycero-3-phosphoethanolamine, 1-oleoyl-L-alpha-lysophosphatidic acid, caprylic acid, D-quinovose, hypoxanthine, L-aspartate, L-fucose, L-glutamate, L-gulonic gamma-lactone, oleic acid, xanthine, and bilirubin were increased in the PLA2G6 mutation group. On the contrary, PLA2G6 mutation carriers had lower levels of 1-hexadecanoyl-2-(9Z-octadecenoyl)-sn-glycero-3-phospho-(1′- rac-glycerol), D-mannose, glyceraldehyde, glycochenodeoxy cholate, phenol, N1-methyl-2-pyridone-5-carboxamide, and *N*-formylmethionine in the negative mode. In the positive mode, His–Glu, L-histidine, and L-palmitoylcarnitine, PC (16:0/16:0) had higher levels in PLA2G6 mutation carriers’ serum, while the concentration of Arg–Ala was higher in healthy subjects.

**TABLE 3:** The 24 metabolites associated with PLA2G6 mutation.

	Compound	Canonical pathway	Mann–Whitney *U*-testing	*t*-test after FDR (q value)	Binary logistic regression	ROC in PLA2G6 mutation		ROC in advanced stage	
						AUC	*P*-value	AUC	*P-*value
1	1-Hexadecanoyl-2-(9Z-octadecenoyl)-sn-glycero-3-phospho-(1′-rac-glycerol)		0.021	0.044	0.067	0.833	0.021		0.606
2	1-Hexadecanoyl-sn-glycero-3-phosphoethanolamine		0.009	0.015	0.06	0.875	0.009		0.439
3	1-Oleoyl-L-alpha-lysophosphatidic acid		0.005	0.016	0.04*	0.903	0.005	0.944	0.039
4	Caprylic acid	Fatty acid biosynthesis	0.003	0.018	0.061	0.931	0.003		1
5	L-Palmitoylcarnitine	Fatty acid degradation	0.007	0.015	0.038*	0.889	0.007		0.606
6	Oleic acid	Biosynthesis of unsaturated fatty acids	0.001	0.010	0.992	1	0.001	0.944	0.039
7	PC(16:0/16:0)	Arachidonic acid metabolism	0.034	0.029	0.083	0.806	0.034		0.439
8	Glycochenodeoxycholate	Primary bile acid biosynthesis	0.009	0.026	0.069	0.875	0.009		0.796
9	D-Mannose	Fructose and mannose metabolism	0.016	0.010	0.11	0.847	0.016		0.606
10	Glyceraldehyde		0.021	0.026	0.044*	0.833	0.021		0.606
11	D-Quinovose		0.001	0.010	0.052	0.958	0.001		0.197
12	L-Fucose	Fructose and mannose metabolism	0.002	0.010	0.055	0.944	0.002		0.197
13	L-Aspartate	Histidine metabolism	0.001	0.010	0.981	0.986	0.001		0.121
14	L-Glutamate	Histidine metabolism	0.004	0.022	0.103	0.917	0.004		0.302
15	L-Histidine	Histidine metabolism	0.012	0.015	0.067	0.861	0.012	1	0.02
16	*N*-Formylmethionine		0.021	0.015	0.031*	0.833	0.021		0.439
17	Arg–Ala		0.043	0.026	0.101	0.792	0.043		0.302
18	His–Glu		0.016	0.015	0.054	0.847	0.016		1
19	Hypoxanthine	Purine metabolism	0.001	0.016	0.977	1	0.001		0.121
20	Xanthine	Purine metabolism	0.004	0.010	0.134	0.917	0.004	0.944	0.039
21	L-Gulonic gamma-lactone	Ascorbate and aldarate metabolism	0.004	0.015	0.112	0.917	0.004		0.071
22	N1-Methyl-2-pyridone-5-carboxamide	Nicotinate and nicotinamide (NAM) metabolism	0.001	0.009	0.983	1	0.001		1
23	Phenol		0.083	0.044	0.239		0.083		0.796
24	Bilirubin	Porphyrin and chlorophyll metabolism	0.068	0.047	0.098		0.068		0.439

Shown are the results of statistical analysis in normalized metabolite levels in PARK14 cases and matched controls, case-control comparison by the Mann–Whitney *U*-test, *t*-test after FDR (false discovery rate) correction, binary logistic regression (with age at exam and gender as covariates), and receiver operator area under the curve (ROC AUC) assessments of predictive ability in diagnosing PLA2G6 mutations and advanced stages (H&Y ≥ 3).

*p < 0.05.

Subsequent annotation through the Kyoto Encyclopedia of Genes and Genomes (KEGG) database revealed the 24 above-mentioned metabolites could be classified into five major Canonical pathways (Table 3, Supplementary Figure 2D, and Supplementary Table 2). Eight of them are related to lipid metabolism pathways, namely, caprylic acid (fatty acid biosynthesis), glycochenodeoxycholate (primary bile acid biosynthesis), L-palmitoylcarnitine (fatty acid degradation), oleic acid (biosynthesis of unsaturated fatty acids), and phosphocholine (16:0/16:0) (arachidonic acid metabolism, linoleic acid metabolism, and glycerophospholipid metabolism) and some unclassified lysophospholipids. Additionally, two metabolites in cofactors and vitamins pathway (L-gulonic gamma-lactone, N1-methyl-2-pyridone-5-carboxamide), four in carbohydrate metabolism pathway (D-mannose, L-fucose, D-quinovose, glyceraldehyde), two in nucleotide metabolism pathway (hypoxanthine, xanthine), three in amino acid metabolism pathway (L-histidine, Arg–Ala, His–Glu), two in both amino acid metabolism and cofactors and vitamins pathway (L-Glutamate, L-Aspartate), and one in porphyrin and chlorophyll metabolism pathway (Bilirubin) were significantly changed in the PLA2G6 Group.

### Association of serum metabolites with clinical features

Because age and gender are predictors of PD (Van Den Eeden et al., 2003) and, given the strong association to fatty acid metabolism, we confirmed each association through multivariate analysis (binary logistic regressions), with age at blood taking, and gender as covariates (Table 3). Four compounds were significantly associated with the PLA2G6 Group, namely glyceraldehyde, L-palmitoylcarnitine, *N*-formylmethionine, and 1-oleoyl-L-alpha-lysophosphatidic acid, in which glyceraldehyde, L-palmitoylcarnitine, and 1-oleoyl-L-alpha-lysophosphatidic acid were found in fatty acid metabolism pathway according to the KEGG database.

**FIGURE 2:**
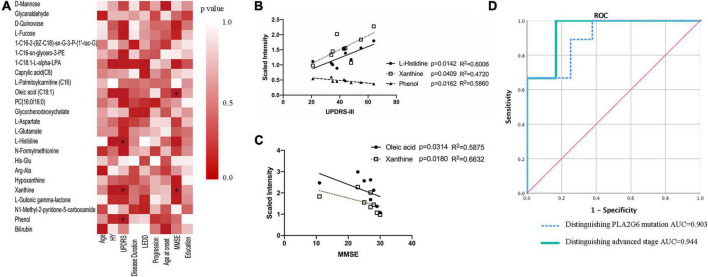
Association of serum metabolites with clinical features. **(A)** Spearman’s correlation analysis between metabolites and clinical features. **(B,C)** Lineal regression of the four compounds with UPDRS-III scores and MMSE scores, respectively. **(D)** ROC curves of a logistic regression model for distinguishing PLA2G6 mutant vs. matched control, advanced stage vs. early stage. *P < 0.05; HY: Hoehn and Yahr stage; LEDD: levodopa equivalent doses; UPDRS, Unified Parkinson’s disease rating scale; MMSE, Mini-Mental State Examination; AUC, area under the curve; ROC, receiver operator curve.

Next, we investigated metabolites associated with clinical features using Spearman’s correlation coefficient. The results showed two metabolites (oleic acid and xanthine) associated with MMSE, and three metabolites associated with UPDRS-III (L-histidine, xanthine, phenol) (*p* < 0.05, Figures 2A–C). No statistically significant correlation was found between any compounds and LEDD.

To assess the ability of each metabolite to determine the diagnosis status, receiver operator curves (ROC) were generated. All previously significantly different metabolites except for phenol and bilirubin showed individual areas under the curve (AUC) >0.79 (Table 3), indicative of good predictive accuracy. The ROC curve of a logistic regression model for distinguishing the PLA2G6 Group from the Healthy Control Group had an AUC of 0.903 (Figure 2D).

Even in the PLA2G6 Group, we observed heterogeneity in the nine patients. Case2, Case6, and Case7 were observed to progress more rapidly (H&Y stage 4–5). Meanwhile, the remaining six cases had an H&Y stage of 2–3. We built ROC models to predict the more advanced subclass of patients in the PLA2G6 Group and four metabolites (1-oleoyl-L-alpha-lysophosphatidic acid, L-histidine, oleic acid, xanthine) from the 23 changed compounds indicated good predictive accuracy for the advanced status of the patients. The resulting AUC values were 0.944 to 1.0 (*p* = 0.039 to 0.02, Table 3). The ROC curve of a logistic regression model for distinguishing early-stage PARK14 from the advanced stage using the above-mentioned four metabolites showed an AUC of 0.944 (Figure 2D).

## Discussion

In the current study, we quantified 50 compounds of metabolites from the serum of all the study subjects, aiming to find potential biomarkers to separate the PLA2G6 Group from the control.

In the OPLS-DA analysis (Supplementary Figure 2B), healthy subjects with single heterozygous mutation (*Single Heterozygous Control Group)* were found in the location next to the mutation negative Healthy Control Group, but on the opposite side of the *PLA2G6 Group*. Moreover, the two patients with single heterozygous PLA2G6 mutation were located exactly between the PLA2G6 Group and the Healthy Control Group. This indicated that the changes in metabolites varied along with the loading of the PLA2G6 mutations.

From the clustering analysis in a heat map in Figure 1, the pattern of metabolites in the two patients in the EOPD Group with single heterozygous PLA2G6 mutation (two light blue columns at the right-most position) was similar to that in the healthy subjects with a single heterozygous mutation (dark blue columns). In addition, the one and only patient with EOPD without any known disease-causing mutation (the one light blue column in the middle) was located near the Healthy Control Group (red columns). Thus, metabolomics analysis showed the ability to distinguish single, biallelic, or no pathogenic mutation in the PLA2G6 gene.

### Lipid metabolism alterations

PLA2G6 encodes calcium-independent phospholipase A2. Our metabolomics data suggest that metabolites in lipid metabolic pathways, particularly fatty acids (fatty acids biosynthesis, elongation, and degradation) and phospholipids (glycerophospholipid metabolism) are highly associated with PLA2G6 mutation status. Of the 24 metabolites that changed significantly in patients’ serum, eight related to lipid metabolism. This makes the lipid metabolism pathway the most frequently affected pathway in the PLA2G6 mutation-related parkinsonism. In addition, oleic acid (C18:1, a monounsaturated long-chain fatty acid) was associated with MMSE scores. Oleic acid and 1-oleoyl-L-alpha-lysophosphatidic acid could also predict the subclass of the more advanced stage in the PLA2G6 Group in ROC models.

According to the research of Mori et al. (2019), the loss of iPLA2-VIA causes the shortening of phospholipid acyl chains in the brain. A higher rate of shorter acyl chains (14:0–14:0 and 14:0–16:1) and a lower proportion of longer acyl chains (18:0–18:0, 18:0–18:1, and 18:0–18:2) were observed in the brain tissues of PLA2G6 knockdown flies in that study. This result of acyl-chain shortening raised the possibility that PLA2G6 preferentially hydrolyzes 14:0 phospholipids, which could eventually affect the metabolism of other classes of lipids and maintain the membrane lipid composition and membrane homeostasis. On the contrary, we detected a lower level of 1-myristoyl-sn-glycero-3-phosphocholine (C14:0) and a higher concentration of compounds with C18:1 fatty acid group (oleic acid, 1-oleoyl-L-alpha-lysophosphatidic acid) in the serum of the PLA2G6 Group. The discordance might originate from the difference in sample selection. The dysfunction of the PLA2G6 enzyme caused relative augmented hydrolysis of longer acyl chains (C18:1 for example) so that the fraction of lipid hydrolysis with longer acyl chains increased in circulation.

In a previous study of 20 patients with *de novo* PD and 20 healthy controls, attenuated levels of C14 to C18 saturated and unsaturated fatty acids and enhanced concentrations of medium-long-chain fatty acids (C6 to C12) were observed in the plasma of the PD cases (Trupp et al., 2014). Also, in another metabolic profiling in the serum of patients with iPD, 1-myristoyl-GPC (14:0) and 2-myristoyl-GPC (14:0), both are lysolipid decreased significantly in the serum of patients with PD (Burte et al., 2017). These findings in iPD were partially consistent with our current study. We also observed an elevated level of caprylic acid (C8:0) and reduced levels of compounds with the C18:0 fatty acid group (stearic acid). Increased caprylic acid (C8:0) level of the PLA2G6 Group was consistent with the studies in serum of patients with PD (Trupp et al., 2014) and restless leg syndrome (RLS; Schulte et al., 2016). In humans, plasma medium-chain fatty acids can be transported into and metabolized by the brain (St-Pierre et al., 2019). Caprylic acid (C8), the main constituent of the medium-chain (C6–C12) triglyceride ketogenic diet, increases the TCA cycle activity of mitochondria within the brain (Wilkins et al., 2016). In the mouse model of PD induced by MPTP, administration of C8 is related to an increase in metabolic activity in striatal mitochondria (Joniec-Maciejak et al., 2018). In PLA2G6 mutation carriers, patients with idiopathic PD as well as patients with RLS enhanced caprylic acid suggest reduced consumption of fatty acids β-oxidation in energy generation due to mitochondrial dysfunction.

However, except for caprylic acid (C8:0), fatty acids with another length of fatty acid chain, from tetracosanoic acid (C24:0) to 2-oxoadipic acid (2-O C6:0) had a lower average level in the PLA2G6 Group, although some species were not statistically significant (Figure 1C). Moreover, we detected a higher concentration of compounds in the C18:1 fatty acid group (oleic acid, 1-oleoyl-L-alpha-lysophosphatidic acid) and C16:0 fatty acid group (1-hexadecanoyl-sn-glycero-3-phosphoethanolamine, L-palmitoylcarnitine, PC 16:0/16:0) in the serum of PLA2G6 Group. After adjusting for age at exam and gender, the significance of changes retained in 1-oleoyl-L-alpha-lysophosphatidic acid and L-palmitoylcarnitine. This inconsistency might be the consequence of the different enzyme activities of phospholipase A2 in idiopathic PD and PLA2G6 pathogenic mutation carriers. We detected lower PLA2 activity in the PLA2G6 Group. But in a study of patients with PD with cognitive impairment, the authors presumed an increased activity or concentration of phospholipase A2 in patients (Zhang et al., 2021).

The differences in changes in fatty acids’ levels are shown in Table 4. Patients with PLA2G6 mutations had elevated lipid compounds in the C18:1 and C16:0 groups. The alteration of lipid metabolism might be the key intermediate process in PLA2G6-related disease that needs further investigation.

**TABLE 4:** Changes in fatty acid metabolism in the serum of individuals with iPD (from the references), PLA2G6 Group (this study), and in the homogenate of PLA2G6 deficient flies (from the reference).

Metabolites	Caprylic acid and derivatives (C8:0)	Myristic acid and derivatives (C14:0)	Palmitic acid and derivatives (C16:0)	Stearic acid and derivatives (C18:0)	Oleic acid and derivatives (C18:1)
iPD	**↑** *	**↓** *	**↓** *	**↓** *	**↓** *
PLA2G6 Group	**↑** *	**↓**	**↑**	**↓**	**↑** *
PLA2G6 deficient flies		**↑** *			**↓** *

iPD, idiopathic Parkinson’s disease; *with statistical significance (*p* < 0.05).

### Other metabolites associated with clinical features

Apart from lipids, several other metabolites were also correlated strongly to MMSE or UPDRS-III scores (Figure 2B).

Xanthine was associated with MMSE and could also predict the more advanced subclass of patients in the PLA2G6 Group in the ROC models. In mammalian tissues under hypoxic conditions, adenosine triphosphate (ATP) degradation results in the accumulation of purine metabolites (Hira et al., 2014). An elevated level of xanthine in the PLA2G6 Group might indicate the increased degeneration of adenosine monophosphate (AMP) and guanosine monophosphate (GMP) during hypoxia. On the contrary, xanthine metabolism was reduced in various studies of PD cases (Schulte et al., 2016; Burte et al., 2017), supporting previous observations in PD LRRK2 mutation carriers (Johansen et al., 2009) and indicating that caffeine absorption may be linked to PD (Hernán et al., 2002). This discordance in our study could be explained by a severer hypoxic condition in the PLA2G6 mutant due to the dysfunction of mitochondria. The difference in caffeine intake in the Asian population might also be an explanation.

L-histidine was another predictor of advanced stage and it showed a significant association with UPDRS-III. Along with L-histidine, the concentration of other compounds in the amino acid metabolism pathway (L-aspartate, L-glutamate) was also increased in the PLA2G6 Group. In a former study on the metabolite panel of iPD (Klatt et al., 2021), the L-histidine level was also elevated in patients. Histamine has a role in neuronal transmission, and elevated metabolite levels have been observed in patients with PD (Nuutinen and Panula, 2010) and increased histamine level is a marker of neuronal damage in PD (Burte et al., 2017).

Phenol was significantly reduced in the PLA2G6 Group, and lower phenol level was associated with severer motor symptoms (higher UPDRS-III scores). The degeneration of phenol was initiated by oxidation catalyzed by monophenol oxidase (mainly tyrosinase). Together with ascorbate oxidase, phenols are oxidized by monophenol oxidase to catechol, then to *O*-benzoquinone. Tyrosinase is widely present in melanocytes and also present at low levels in the brain (Carballo-Carbajal et al., 2019). In dopaminergic neurons of the substantia nigra (SN), neuromelanin (NM) is synthesized from tyrosine or levo-dopamine (L-Dopa) through a series of oxidation processes that are catalyzed by tyrosinase (Wakamatsu et al., 2019). NM accumulation in the brain increases with age, but is markedly decreased in neurodegenerative diseases such as PD (Zecca et al., 2006). Overexpression of human tyrosinase in rat SN results in age-dependent NM accumulation within nigral dopaminergic neurons and PD phenotype (Carballo-Carbajal et al., 2019). The lower concentration of Phenol in the PLA2G6 Group in our study may indicate the hyperfunction of tyrosinase in PLA2G6 mutation carriers, which can also lead to abnormality in the metabolism of dopamine and NM.

Despite strong associations with the PLA2G6 Group, there were still some metabolites that had an impact on pathway analysis but could not be correlated to clinical measurements of disease severity (namely MMSE and UPDRS-III).

In the porphyrin metabolic pathway, higher serum bilirubin contents have been observed in patients with PD, essential tremor (ET) (Albillos et al., 2021), and PLA2G6 mutations (our study). This increase in bilirubin contents might be due to heme oxygenase overexpression, which might be a compensatory response to oxidative stress arising from enzyme upregulation of heme oxygenase in the SN (Moccia et al., 2015).

In the nicotinate and nicotinamide (NAM) metabolic pathway, we observed a lower concentration of N1-methyl-2-pyridone-5-carboxamide (2PY) in the PLA2G6 Group. N1-methyl-2-pyridone-5-carboxamide (2PY) is the final product of NAM in NADP + (NAM adenine dinucleotide phosphate) degeneration (Lenglet et al., 2016). NAD + metabolism plays a crucial role in PD pathogenesis in a number of PD-associated disease models that present mitochondrial defects (Gasperi et al., 2019). As Lehmann et al. reported (Lehmann et al., 2017), in the PINK1 mutant flies, they detected significant reductions in the level of NAD+, as well as the NAD + salvage metabolite NAM ribonucleotide (NMN) and NAD+ precursor NAM riboside (NR). Defects in mitophagy caused by mutations in PINK1 lead to the disruption of mitochondrial bioenergetics and thereby increased the redox state of the complex I substrate NAM adenine dinucleotide (NAD+) (Tufi et al., 2014). Given that the PLA2G6 mutation knockin mouse model displayed mitochondrial dysfunction and reduced activity of mitochondrial Complex I (Chiu et al., 2019), the decreased level of 2PY (a final catabolite of NAD+) in subjects of the PLA2G6 Group is quite understandable.

We also detected the level of *N*-Formylmethionine decreased in patients with PARK14. *N*-Formylmethionine is the initiation amino acid for intra-mitochondrial protein translation (Tucker et al., 2011). One study by Cai et al. (2021) found that mutations in mitochondria DNA (mtDNA) modulate the level of circulating *N*-formylmethionine after adjusting bacterial exposure. In a further 11,966 individuals, *N*-formylmethionine levels contributed to all-cause mortality and the disease risk of several common age-related diseases (Cai et al., 2021). PLA2G6 mutation of PARK14 (D331Y) knockin mouse model displayed mitochondrial dysfunction and upregulated ROS production (Chiu et al., 2019). Thus, the variation of circulation N-formylmethionine concentration in our study might be the consequence of altered mitochondria function.

The findings of some other metabolites, such as D-mannose, D-quinovose, L-fucose, and L-gulonic gamma-lactone, which locate in pathways with extremely low impact in KEGG analysis, are more difficult to interpret. Because of the limited sample size, we cannot rule out the possibility of false positive findings in this metabolomic study.

PLA2G6 is an intracellular phospholipase. Assaying the activity of phospholipases A2 (PLA2s) has been challenging because they are water-soluble enzymes acting on water-insoluble phospholipid substrates (Mouchlis et al., 2019). The enzymatic activity of calcium-independent phospholipase-A2 could only be measured in cell lysates. Skin biopsy for skin fibroblast is an invasive procedure for patients. Moreover, whether PLA2G6 missense mutations affect enzymatic activity in fibroblast or not is still controversial (Engel et al., 2010) and the results were often unstable. Serum metabolite profiling provides a relatively novel tool to query an easily accessible biospecimen (i.e., serum) for the reflections of pathophysiologic processes and potential biomarkers. Metabolomics examination, especially lipid species profiling, provides the possibility of finding an easily measured circulating biomarker for diagnosing patients with PLA2G6 mutations and evaluating patients’ cognition and disease severity. In disease-modifying treatment, the biomarkers can also be used as indexes in monitoring disease progression and treatment success.

### Limitations

The strength of our conclusion is tempered by some discrepancies, namely, the limited sample size and the cross-sectional design. Because of the extremely low sample size (only five subjects donated their fibroblasts), we cannot analyze the correlation of disease phenotype and metabolites with enzyme activity for now. We also lack enough patients with mutation-negative PD with matched age and gender as EOPD control. Thus, further studies including larger sample numbers and a longitudinal follow-up might help to confirm these results. In addition, this study requires further validation of physiological and pathological processes.

## Conclusion

In conclusion, long-chain fatty acid metabolism-derived metabolites (like oleic acid), xanthine, L-histidine, and phenol are related to the disease severity of PLA2G6 mutation-associated dystonia–parkinsonism (PARK14). Enzymatic changes related to phospholipids with long-chain fatty acids are suggested to be a key process in PARK14 etiology. Serum metabolomic changes may provide essential information for the selection of potential biomarkers for PLA2G6 mutation status. However, further research studies using larger longitudinal cohorts are necessary to confirm our results.

## Data availability statement

The original contributions presented in the study are publicly available. This data can be found in MetaboLights as MTBLS4391 at the following link: https://www.ebi.ac.uk/metabolights/MTBLS4391.

## Ethics statement

The studies involving human participants were reviewed and approved by the Ethics Committee of Huashan Hospital of Fudan University. The patients/participants provided their written informed consent to participate in this study.

## Author contributions

Y-MS, W-YW, and JW: conceptualization. M-ML, FL: methodology. CC: data analysis and draft writing. FL, F-TL, and S-SL: draft reviewing and editing. All authors have read and agreed to the published version of the manuscript.

## Footnotes

1
http://www.ncbi.nlm.nih.gov/clinvar

2
http://proteowizard.sourceforge.net
