# Supplementary material for: Serum metabolomic characterization of PLA2G6-associated dystonia–parkinsonism: A case-control biomarker study
Source: Front Neurosci. 2022 Aug 11;16:879548. doi: 10.3389/fnins.2022.879548 (PMC9406281; doi:10.3389/fnins.2022.879548)
Supplement: Supplementary file 1 [file Data_Sheet_1.docx]

Supplementary Material

# Supplementary Table S1: The Parkinson’s disease related genes tested in the cohort

| PSEN1 | ATM | ATP7B | CP | TOR1A | FXN | FTL | GBA |
| --- | --- | --- | --- | --- | --- | --- | --- |
| GCH1 | KCNA1 | PEX7 | PRNP | ATXN1 | ATXN7 | SCN4A | SNCA |
| TTPA | IL1B | ADH1C | CACNB4 | CYP27A1 | DRD2 | DRD3 | GPNMB |
| FBXO7 | PHYH | SIL1 | SLC6A3 | SPPL2B | PNKD | CCDC88C | TENM4 |
| GIGYF2 | TMEM240 | CACNA1A | SLC39A14 | BEAN1 | FAM47E | SQSTM1 | GNAL |
| ITPR1 | TBP | ADCY5 | C9orf72 | DNAJC6 | DGKQ | DLG2 | DNMT1 |
| GRID2 | MR1 | IFRD1 | ATN1 | EEF2 | FMR1 | GRN | HTT |
| HLA-DQB1 | HPCA | PDGFB | PDGFRB | POLG | PRKCG | RIT2 | ATXN2 |
| SPR | NKX2-1 | PLA2G6 | ITGA8 | CASK | PRKRA | SYNJ1 | SGCE |
| RAB29 | DCTN1 | FGF14 | SLC1A3 | UCHL1 | BST1 | COL6A3 | PRKN |
| TAF1 | XPR1 | FUS | MECP2 | ATXN3 | ISG15 | GAK | SCARB2 |
| ATP6AP2 | BCKDK | MMP16 | TUBB4A | NOP56 | SLC2A1 | PNPLA6 | SLC20A2 |
| AFG3L2 | SPTBN2 | WDR45 | VCP | PARK7 | TARDBP | CIZ1 | SCAP |
| STK39 | ATXN10 | HTRA2 | TBK1 | USP25 | UBQLN2 | CHMP2B | SACS |
| INPP5F | SETX | VPS13A | DNAJC13 | COQ2 | NMD3 | CHCHD2 | MAPT |
| VPS13C | ANO10 | THAP1 | VPS35 | SLC30A10 | CACNA1G | FGF20 | MCCC1 |
| JPH3 | SIPA1L2 | SIPA1L2 | SCN2A | XK | ELOVL5 | TWNK | ATP13A2 |
| ELOVL4 | NUCKS1 | DDRGK1 | L1CAM | FA2H | PDYN | KCTD17 | PANK2 |
| COASY | RAI1 | TMEM163 | ANO3 | TMEM175 | PINK1 | STX1B | ACMSD |
| PRRT2 | SYT11 | ATP1A3 | KCNC2 | SNX14 | RAB39B | KCND3 | TTBK2 |
| APTX | PPP2R2B | TMEM229B | EIF4G1 | LRRK2 | TGM6 | TH | ABCB7 |
| ACAT1 | APOE | ATP2B3 | ATP7A | ERCC8 | TPP1 | CSTB | DCC |
| DDC | TIMM8A | DRD5 | ECM1 | ERCC6 | GCDH | GLB1 | GLUD2 |
| GRM1 | HPRT1 | HSD17B4 | KCNA2 | KCNMA1 | KCNS2 | MC1R | MEIS1 |
| MRE11 | MTTP | MUT | NDUFS4 | NOS3 | NPC1 | NR4A2 | PCCA |
| PCCB | MAP2K5 | PTPRD | PTS | QDPR | RAD51 | SORT1 | SCN1A |
| SLC1A2 | SLC6A4 | SLC16A2 | SNCB | SURF1 | VAMP1 | GOSR2 | SNCAIP |
| ZNF592 | RUBCN | STUB1 | NPC2 | KIF1C | WASF3 | PDE10A | PNKP |
| SYNE1 | PIK3R5 | COQ8A | TOX3 | TOR1B | WWOX | TREM2 | TRPM7 |
| MTPAP | CWF19L1 | TDP1 | COQ8A | GJD2 | GBA2 | HS1BP3 | IPPK |
| USP46 | DCAF17 | SPG11 | SLC19A3 | C19orf12 | LINGO1 | ATCAY | MARS2 |
| BTBD9 | PACRG | PRICKLE1 | KCTD7 | SYT14 | HAPLN4 |  |  |

**Supplementary Table S2:** **Parameters corresponding to the circles of KEGG pathways**

**Figure S2d** represent the pathways having relevance in the pathway enrichment analysis. Each pathway can be localized in the figure according to their impact and –log(p) value.

| **pathway** | **match status** | **p** | **-log(p)** | **Holm p** | **FDR** | **impact** | **details** |
| --- | --- | --- | --- | --- | --- | --- | --- |
| D-Glutamine and D-glutamate metabolism | 1/6 | 0.013729 | 1.8623 | 0.28832 | 0.02569 | 0.5 | KEGG  SMP |
| Alanine, aspartate and glutamate metabolism | 2/28 | 0.003938 | 2.4047 | 0.10239 | 0.015752 | 0.42068 | KEGG  SMP |
| Histidine metabolism | 3/16 | 0.0021206 | 2.6735 | 0.057256 | 0.01131 | 0.22131 | KEGG  SMP |
| Lysine degradation | 2/25 | 0.26409 | 0.57824 | 1 | 0.313 | 0.14085 | KEGG  SMP |
| Arginine biosynthesis | 2/14 | 0.003938 | 2.4047 | 0.10239 | 0.015752 | 0.11675 | KEGG |
| Glycerophospholipid metabolism | 2/36 | 0.044651 | 1.3502 | 0.35721 | 0.057153 | 0.11182 | KEGG |
| Cysteine and methionine metabolism | 1/33 | 0.39502 | 0.40338 | 1 | 0.45145 | 0.10446 | KEGG  SMP |
| Arginine and proline metabolism | 1/38 | 0.013729 | 1.8623 | 0.28832 | 0.02569 | 0.086 | KEGG  SMP |
| Porphyrin and chlorophyll metabolism | 2/30 | 0.015253 | 1.8166 | 0.28832 | 0.02569 | 0.05288 | KEGG |
| Purine metabolism | 3/65 | 0.0049864 | 2.3022 | 0.11967 | 0.016146 | 0.02868 | KEGG  SMP |
| Glutathione metabolism | 1/28 | 0.013729 | 1.8623 | 0.28832 | 0.02569 | 0.01966 | KEGG  SMP |
| Pyrimidine metabolism | 1/39 | 0.87068 | 0.06014 | 1 | 0.87068 | 0.01584 | KEGG  SMP |
| Fatty acid biosynthesis | 2/47 | 0.62763 | 0.2023 | 1 | 0.69256 | 0.01473 | KEGG  SMP |
| Primary bile acid biosynthesis | 1/46 | 0.0145 | 1.8386 | 0.28832 | 0.02569 | 0.00977 | KEGG  SMP |
| Nicotinate and nicotinamide metabolism | 2/15 | 5.07E-05 | 4.2954 | 0.0016209 | 0.0016209 | 0 | KEGG  SMP |
| beta-Alanine metabolism | 2/21 | 1.58E-03 | 2.8027 | 0.048825 | 0.01131 | 0 | KEGG  SMP |
| Fructose and mannose metabolism | 1/20 | 1.63E-03 | 2.7879 | 0.04889 | 0.01131 | 0 | KEGG  SMP |
| Amino sugar and nucleotide sugar metabolism | 1/37 | 0.0016297 | 2.7879 | 0.04889 | 0.01131 | 0 | KEGG  SMP |
| Pantothenate and CoA biosynthesis | 1/19 | 0.0019119 | 2.7185 | 0.053534 | 0.01131 | 0 | KEGG  SMP |
| Aminoacyl-tRNA biosynthesis | 4/48 | 0.0050455 | 2.2971 | 0.11967 | 0.016146 | 0 | KEGG |
| Ascorbate and aldarate metabolism | 1/8 | 0.007031 | 2.153 | 0.15468 | 0.020454 | 0 | KEGG |
| Glyoxylate and dicarboxylate metabolism | 1/32 | 0.013729 | 1.8623 | 0.28832 | 0.02569 | 0 | KEGG |
| Butanoate metabolism | 1/15 | 0.013729 | 1.8623 | 0.28832 | 0.02569 | 0 | KEGG  SMP |
| Nitrogen metabolism | 1/6 | 0.013729 | 1.8623 | 0.28832 | 0.02569 | 0 | KEGG  SMP |
| Biosynthesis of unsaturated fatty acids | 3/36 | 0.018937 | 1.7227 | 0.28832 | 0.026682 | 0 | KEGG |
| Fatty acid degradation | 2/39 | 0.019371 | 1.7128 | 0.28832 | 0.026682 | 0 | KEGG  SMP |
| Arachidonic acid metabolism | 1/36 | 0.020011 | 1.6987 | 0.28832 | 0.026682 | 0 | KEGG  SMP |
| Linoleic acid metabolism | 1/5 | 0.020011 | 1.6987 | 0.28832 | 0.026682 | 0 | KEGG  SMP |
| alpha-Linolenic acid metabolism | 1/13 | 0.020011 | 1.6987 | 0.28832 | 0.026682 | 0 | KEGG  SMP |
| Tryptophan metabolism | 1/41 | 0.21208 | 0.67349 | 1 | 0.26102 | 0 | KEGG  SMP |
| Vitamin B6 metabolism | 1/9 | 0.71269 | 0.1471 | 1 | 0.76021 | 0 | KEGG  SMP |
| Fatty acid elongation | 1/39 | 0.7626 | 0.1177 | 1 | 0.7872 | 0 | KEGG  SMP |

The pathways having relevance in the pathway enrichment analysis was represent in **Supplementary Figure S2**. Each pathway can be localized in the figure according to their impact and –log(p) value.

**Supplementary Figure S1: Enzyme activity of fibroblasts**

**
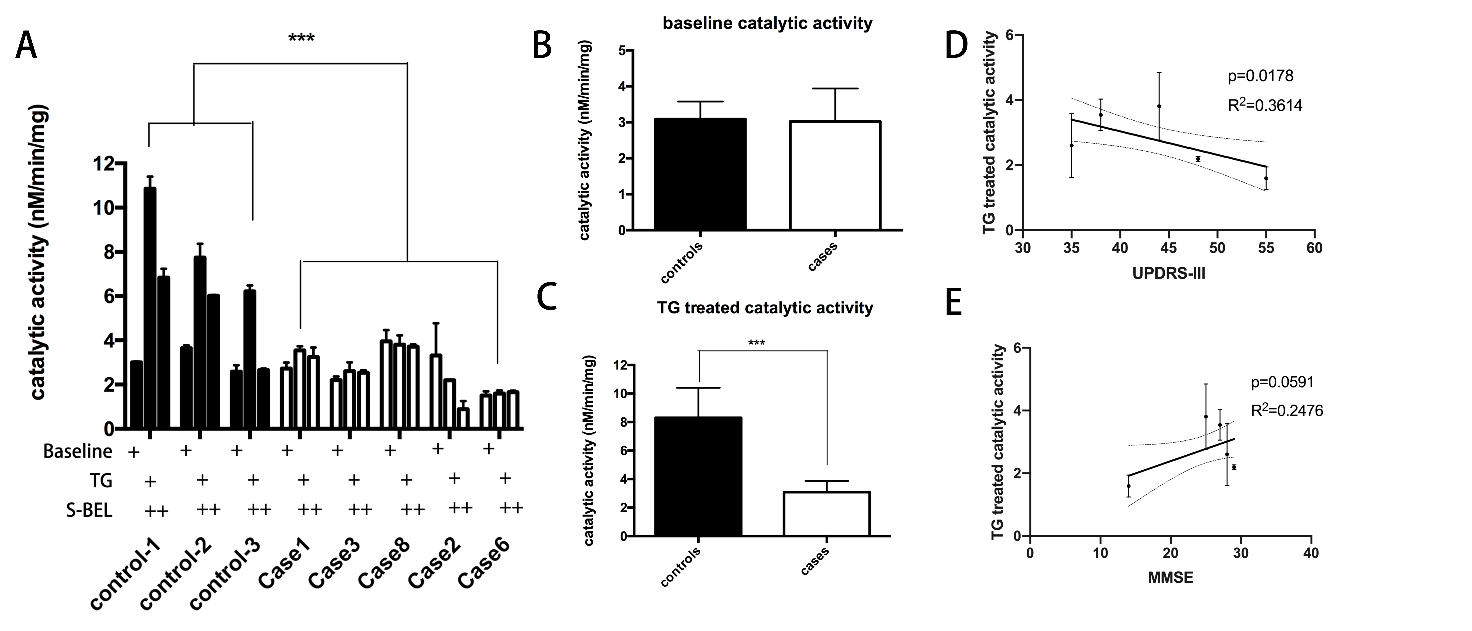
**

**Supplementary Figure S1:** **(A)** Summary data show activation of PLA2g6 by TG-induced depletion of Ca^2+^ stores in intact hPSF from individual control and PLA2G6-related Dystonia-Parkinsonism (PARK14) cases: summary data for each patient show average activity (±SEM) from 3 repetitions in basal conditions, after activation by TG (5 μM for 10 minutes), and after inhibition with (S)-BEL. *** p<0.0001. **(B, C)** Ca^2+^ store-dependent activation of PLA2g6 is significantly impaired in fibroblasts derived from PARK14 patients compared with the control group, though the baseline catalytic activity of PLA2g6 remained unchanged. *** p<0.0001.

**Supplementary Figure S2: Overview of metabolites and KEGG pathway enrichment analysis**

**
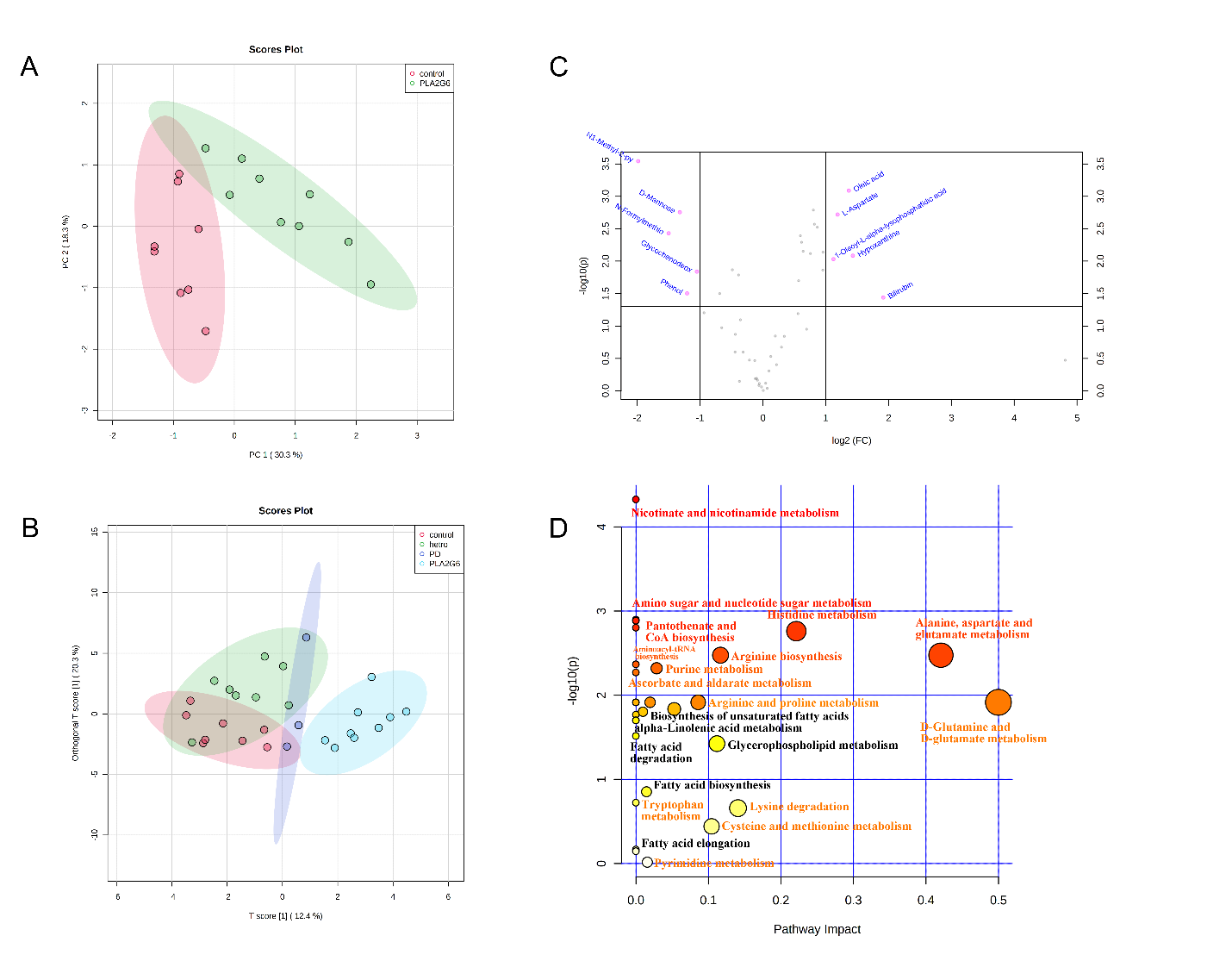
**

**Supplementary Figure S2:**: **(A)** Principal component analysis (PCA) score plots of metabolic profiles in PLA2G6 Group (green dots, PLA2G6) and Healthy Control Group (red dots, control); **(B)** Orthogonal Projections to Latent Structures Discriminant Analysis (OPLS-DA) score plots of metabolic profiles in PLA2G6 Gdroup (blue dots, PLA2G6), Healthy Control Group (red dots, control), single heterozygous PD Group (purple dots, PD) and single heterozygous control Group (green dots, hetro); **(C)** Volcano plot for PLA2G6 Group versus Healthy Control Group; **(D)** Graphical view of the pathway impact on the differences between PLA2G6 Group and Healthy Control Group. Detailed pathway enrichment analysis was shown in **Supplementary Table S2**.

**Supplementary Figure S3: Example of feature intensity distribution before and after data normalization by range scaling.**

**
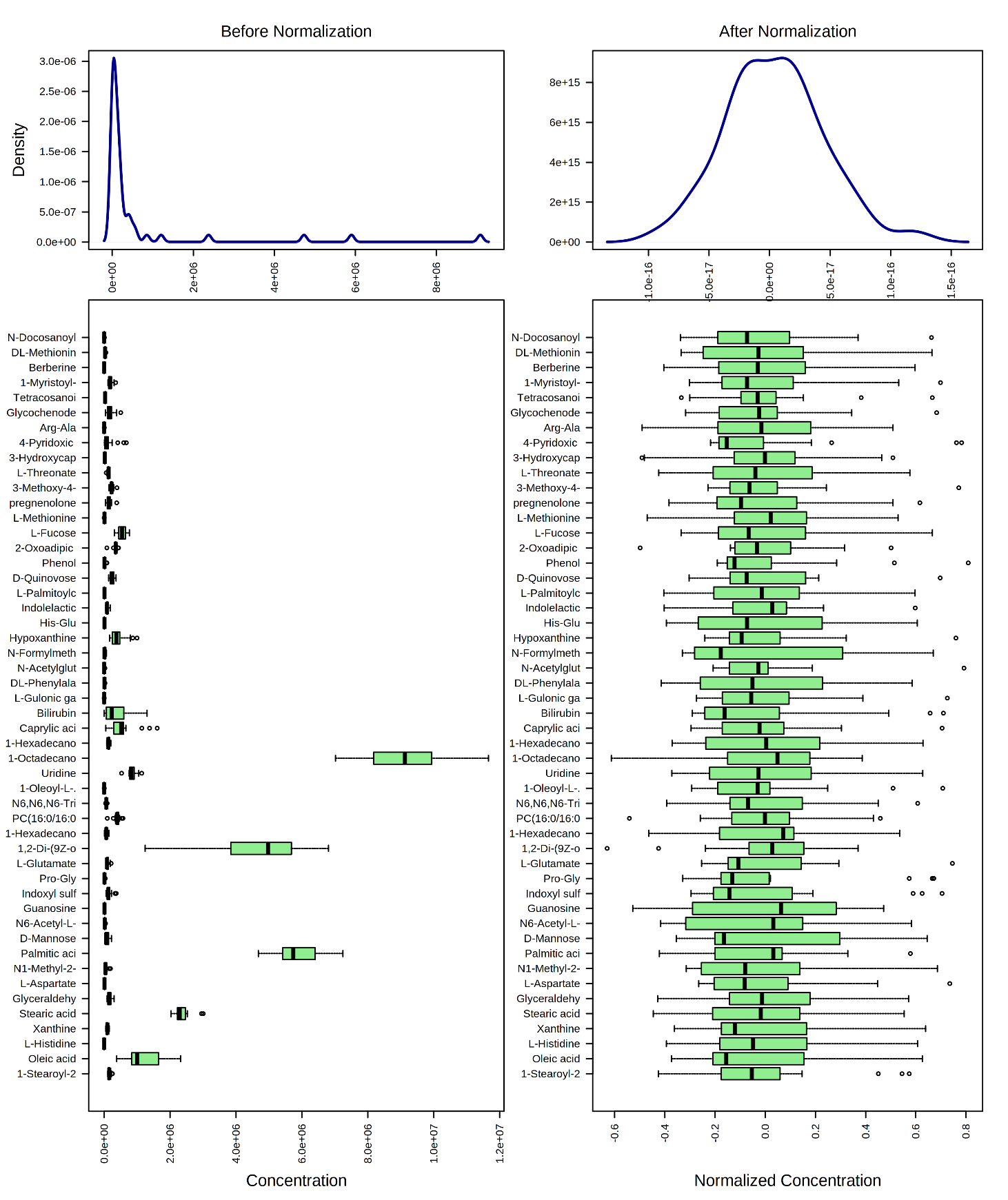
**
